# Federated Learning Framework for Real-Time Activity and Context Monitoring Using Edge Devices

**DOI:** 10.3390/s25041266

**Published:** 2025-02-19

**Authors:** Rania A. Alharbey, Faisal Jamil

**Affiliations:** 1 Department of Mathematics, Faculty of Science, King Abdulaziz University, Jeddah 21589, Saudi Arabia; 2 School of Computing, Engineering and Intelligent Systems, Ulster University, Derry-Londonderry BT48 7JL, Northern Ireland, UK; f.jamil@ulster.ac.uk

**Keywords:** federated learning, elderly care monitoring, altitude detection, location tracking, contextual monitoring

## Abstract

With the increasing need for effective elderly care solutions, this paper presents a novel federated learning-based system that uses smartphones as edge devices to monitor and enhance elderly care in real-time. In this system, elderly individuals carry smartphones equipped with Inertial Measurement Unit (IMU) sensors, including an accelerometer for activity recognition, a barometer for altitude detection, and a combination of the accelerometer, gyrometer, and magnetometer for location tracking. The smartphones continuously collect real-time data as the elderly individuals go about their daily routines. These data are processed locally on each device to train personalized models for activity recognition and contextual monitoring. The locally trained models are then sent to a federated server, where the FedAvg algorithm is used to aggregate model parameters, creating an improved global model. This aggregated model is subsequently distributed back to the smartphones, enhancing their activity recognition capabilities. In addition to model updates, information on the users’ location, altitude, and context is sent to the server to enable the continuous monitoring and tracking of the elderly. By integrating activity recognition with location and altitude data, the system provides a comprehensive framework for tracking and supporting the well-being of elderly individuals across diverse environments. This approach offers a scalable and efficient solution for elderly care, contributing to enhanced safety and overall quality of life.

## 1. Introduction

The global population of elderly individuals is growing rapidly, leading to increased demand for efficient monitoring and care solutions in order to improve their safety and quality of life [1]. One of the critical aspects of elderly care is real-time monitoring, which enables caregivers and healthcare professionals to track the physical activity, location, and well-being of elderly individuals, particularly those living in care facilities or independently at home [2,3]. Technological advancements, such as the proliferation of smartphones and wearable devices, have introduced new possibilities for health monitoring, making it possible to collect data from various sensors in real-time and process it for valuable insights [4,5]. Smartphones, which are widely used across all age groups, have proven to be effective tools for monitoring individuals, as they are equipped with several sensors capable of capturing critical data, including activity levels, location, and environmental factors [6]. In this context, smartphones can serve as edge devices for continuous monitoring in elderly care systems, where real-time data collection and analysis can offer significant benefits [7,8]. Specifically, smartphones are equipped with Inertial Measurement Unit (IMU) sensors, such as accelerometers, gyrometers, and magnetometers, which can be used to detect movements and provide detailed information about a person’s physical activities and location [9].

Federated learning has emerged as a powerful technique for training machine learning models in a decentralized manner, allowing data to remain on local devices while model updates are shared with a central server [10]. This approach is particularly useful in cases where data privacy is a concern, as it ensures that raw data are not transmitted to the server [11,12]. However, in the context of elderly care, continuous monitoring often requires certain data, such as location and altitude information, to be transmitted to the server for effective tracking and oversight [13]. Despite this, federated learning still plays a crucial role in enhancing the performance of activity recognition models across multiple devices without the need for centralizing all the raw data [14,15].

This paper proposes a novel system for elderly care that utilizes federated learning with smartphones as edge devices, where elderly individuals carry smartphones equipped with IMU sensors for continuous data collection. The proposed system focuses on monitoring the elderly through three key components—activity recognition, altitude detection, and location tracking, all of which are critical for understanding the day-to-day activities and safety of elderly individuals [16]. By integrating federated learning into this system, we can improve the accuracy of activity recognition models while simultaneously tracking the users’ altitude and location data in real-time [17,18].

Activity recognition plays a crucial role in understanding the physical well-being of elderly individuals, as changes in activity patterns can often indicate health issues such as falls, mobility problems, or physical inactivity [19]. IMU sensors, particularly accelerometers, have been extensively used to detect human activities such as walking, sitting, and standing, and they provide an effective solution for non-intrusive monitoring [20]. Additionally, gyrometers and magnetometers complement accelerometers by providing information about the orientation and movement of the device, which helps in refining the detection of complex activities and distinguishing between different types of movements [21].

Altitude detection, another important aspect of elderly care, can be particularly relevant for individuals living in multi-story buildings or care facilities, where changes in altitude may indicate movement between floors [22]. The barometer sensor in smartphones measures atmospheric pressure and can be used to estimate changes in altitude with high accuracy. This capability is critical for tracking the location and movement of elderly individuals within such environments, ensuring that caregivers are aware of their whereabouts.

Location tracking is an essential feature for ensuring the safety and well-being of elderly individuals, especially for those who may suffer from cognitive impairments or wander off from their designated locations [23]. By leveraging the combination of accelerometers, gyrometers, and magnetometers, along with GPS when available, smartphones can provide reliable location information, even in environments where GPS signals may be weak, such as indoors. This capability allows for the continuous tracking of elderly individuals in care facilities or during their daily routines, enabling real-time alerts and interventions when necessary [24].

The use of federated learning in this system offers several advantages over traditional centralized approaches. First, federated learning allows the system to continually improve the accuracy of activity recognition models by aggregating updates from multiple devices while reducing the computational and communication burden on the central server. The FedAvg algorithm, which averages the locally trained models from each smartphone, ensures that the global model benefits from the diversity of data collected across different users and environments. This process allows the model to generalize better to different scenarios, improving the system’s overall performance.

The proposed system enables continuous model updates without centralized data storage, minimizing the risk of data breaches and allowing seamless integration of new data without disrupting the monitoring process. This holistic approach captures elderly individuals’ activity patterns, health status, and location, providing caregivers with actionable insights. By incorporating real-time location and altitude data, the system offers a comprehensive framework for monitoring movements and behaviors, enabling rapid responses to emergencies. For example, sudden altitude changes coupled with inactivity could indicate a fall, while location tracking helps prevent wandering or disorientation.

The notable contributions of the proposed study are as follows:

- Multi-Sensor Data Integration: The system combines activity recognition, location tracking, and altitude detection using smartphone IMU sensors for real-time elderly monitoring.
- Federated Learning for Activity Recognition: Federated learning with FedAvg enhances activity recognition accuracy without centralizing raw data.
- Real-Time Elderly Tracking: The system provides the real-time monitoring of location, altitude, and context, enabling rapid emergency response.
- Scalable and Non-Intrusive Solution: The use of smartphones as edge devices offers a scalable, non-intrusive monitoring system for elderly care.

The rest of the paper is divided into the following sections. Section 2 presents the related works. Section 3 details the Federated Learning-Based Elderly Monitoring System Using Smartphone Sensors, covering data collection, local model training, and federated learning aggregation. Section 4 presents the experimental results and discussion, including performance evaluation, accuracy improvements, model compression effects, and scalability analysis. Finally, Section 5 concludes the paper with a summary of the findings and future research directions.

## 2. Related Work

The monitoring of elderly individuals for health and safety has garnered significant research attention due to the growing aging population and the increasing demand for non-intrusive and scalable monitoring solutions. Various approaches have been proposed for activity recognition, location tracking, real-time elderly care, and network monitoring, leveraging different technologies and methodologies [25,26,27,28,29,30,31,32,33]. A widely adopted technique in elderly monitoring is wearable-based activity recognition, which, despite its effectiveness, often lacks scalability and comfort for long-term deployment in elderly care settings. Given the ubiquity of smartphones equipped with advanced sensors, they have emerged as a viable alternative for activity recognition and elderly care applications [22].

### 2.1. Activity Recognition Using Smartphone Sensors

Inertial Measurement Unit sensors, including accelerometers, gyroscopes, and magnetometers, have been extensively utilized for activity recognition, enabling the detection of common human movements such as walking, sitting, and running [34,35,36,37,38,39]. Given their integration into smartphones, these sensors provide a convenient platform for continuous monitoring. Prior studies, such as [40], have demonstrated the feasibility of using smartphone-based accelerometers for human activity recognition, underscoring their potential in real-world health monitoring systems. However, most existing approaches rely on centralized data collection, which poses challenges related to scalability and flexibility.

### 2.2. Federated Learning in Elderly Monitoring

Federated learning has gained traction as a decentralized machine learning paradigm, particularly in privacy-sensitive applications [41]. This technique enables devices to train models locally and share only model updates with a central server, thereby preserving data privacy [42]. Early implementations of federated learning focused on mobile environments, with Google pioneering its use in predictive text applications without requiring centralized data storage [43]. Subsequent research, including [44], expanded federated learning to broader mobile and IoT applications, demonstrating its potential for elderly monitoring systems that demand scalability [45].

In healthcare, federated learning has been explored for collaborative medical diagnostics, allowing multiple institutions to train models on sensitive patient data without directly sharing information [46]. In another study, the researcher applied federated learning for medical image classification, showing that decentralized learning improves model performance while maintaining data privacy [47]. While privacy remains a key advantage, federated learning in the proposed system is primarily leveraged to enhance activity recognition accuracy by aggregating models from multiple devices [48].

### 2.3. Location Tracking for Elderly Care

Location tracking is crucial for elderly individuals, particularly those with cognitive impairments, as it aids in mitigating risks associated with wandering and disorientation [49]. Conventional GPS-based tracking systems often suffer from poor accuracy indoors due to signal attenuation [50]. To address this limitation, researchers have incorporated IMU sensors, such as magnetometers and gyroscopes, to complement GPS data, enhancing indoor localization accuracy. Ref. [51] demonstrated that fusing accelerometer and magnetometer data improves positioning accuracy in environments with weak GPS signals, a strategy that aligns with the multi-sensor approach adopted in the proposed system [45].

### 2.4. Altitude Detection for Multi-Level Tracking

Barometric sensors have been widely employed for altitude detection in multi-story buildings and uneven terrain [52]. These sensors, by measuring atmospheric pressure variations, provide insights into vertical movement, enabling systems to track floor transitions in elderly care facilities [52]. Ref. [53] utilized smartphone barometer readings to monitor the elevation of elderly individuals in care homes, ensuring caregivers remain informed about their precise location [54]. The integration of altitude detection into real-time monitoring systems enhances caregiver awareness, adding an extra layer of safety for elderly individuals [55].

### 2.5. Existing Integrated Systems and Limitations

The combination of activity recognition, location tracking, and altitude detection has been investigated across multiple domains. However, few studies have integrated these components into a unified framework tailored for elderly care [56]. In another study, the author proposed a smartphone-based system for monitoring elderly individuals’ activities and locations but lacked altitude detection capabilities [57]. Similarly, ref. [58] introduced a wearable-smartphone hybrid system for activity recognition and location tracking, yet it lacked real-time federated aggregation. The novelty of the proposed work lies in the seamless integration of federated learning with activity recognition, location tracking, and altitude detection, creating a scalable and real-time monitoring framework for elderly individuals.

Federated learning in elderly care remains an emerging research area, with only a few studies exploring its full potential. Ref. [59] applied federated learning for fall detection using wearable sensors, highlighting its ability to enhance accuracy while accommodating diverse data distributions. However, their approach was predominantly focused on wearable devices rather than smartphone-based systems, thereby limiting scalability [60]. The proposed system extends this work by leveraging federated learning in a smartphone-based environment, which is inherently more accessible and widely adopted in elderly care settings [61].

None of the existing systems provide a fully integrated federated learning-based framework that combines activity recognition, location tracking, and altitude detection while maintaining scalability and privacy as shown in Table 1. Most approaches rely on centralized processing, leading to privacy risks, or require additional infrastructure such as BLE beacons or RFID, limiting real-world deployment. The proposed model leverages federated learning to ensure privacy-preserving, real-time monitoring while integrating multi-sensor data for accurate and scalable elderly care. Additionally, model compression minimizes the communication overhead, ensuring efficient deployment in resource-constrained environments.

## 3. Federated Learning-Based Elderly Monitoring System Using Smartphone Sensors

This study builds upon prior advancements in federated learning, activity recognition, and indoor localization, employing methodologies that enhance human activity monitoring and smartphone-based tracking. The PDR-BLE compensation mechanism has demonstrated improvements in indoor localization through sensor fusion, particularly by utilizing accelerometer data for activity detection [64]. Furthermore, an optimal ensemble scheme for human activity recognition (HAR) and floor detection was developed using AutoML, thereby enhancing HAR accuracy through smartphone sensor data [65]. Similarly, a fusion-based localization approach was proposed for tracking smartphone users in multistory buildings, integrating advanced methodologies to address the complexities of indoor environments [66]. Recent advancements in evolutionary-enhanced particle filters have focused on the rapid tracking of smartphone users in hazardous scenarios, serving as an inspiration for our approach, which integrates multiple sensor data streams to improve recognition accuracy [67]. Additionally, Jamil et al. (2024) introduced a hybrid federated learning strategy that optimizes model aggregation across edge devices, thereby enhancing activity recognition [68]. The Swarm Learning Empowered Federated Deep Learning framework has further contributed to optimizing communication efficiency in federated learning, a key aspect incorporated into our elderly care system [69].

**Figure 1:**
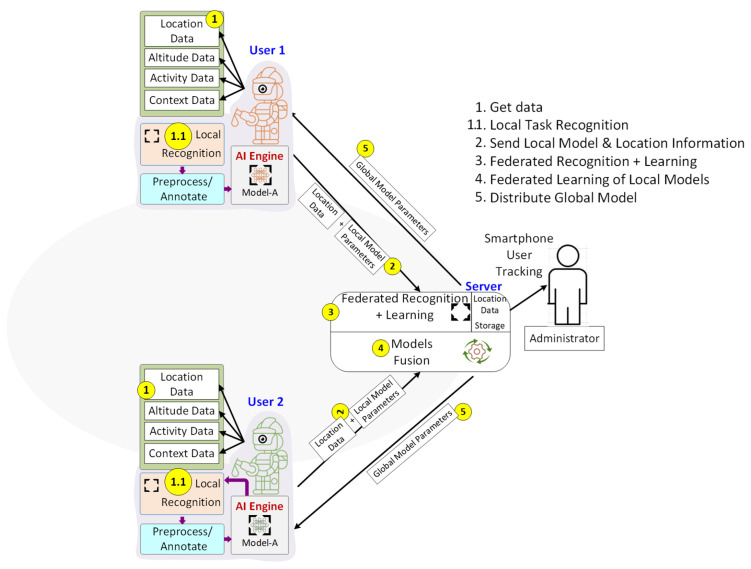
Federated learning-based elderly monitoring system architecture.

The proposed federated learning-based elderly monitoring system employs smartphones as edge devices as illustrated in Figure 1. The system continuously collects real-time data from built-in smartphone sensors for activity recognition, location tracking, altitude estimation, and contextual awareness. Federated learning ensures that models are trained locally, with periodic updates aggregated at a central server, enhancing performance without sharing raw data.

The process involves sensor-based data collection, local preprocessing, and activity classification using personalized models. These locally trained models are periodically updated and merged using the Federated Averaging (FedAvg) algorithm to create a global model, which is redistributed to all devices for continuous refinement. This approach supports real-time emergency detection, activity monitoring, and user tracking while maintaining privacy through decentralized learning, making it a scalable and efficient solution for elderly care.

The following sections detail the system architecture, covering sensor-based data collection, local activity recognition, the federated learning process, global model fusion, and continuous monitoring strategies. The system leverages smartphone sensors to collect real-time user data, preprocess them locally, and classify activities using machine learning models. These models are periodically updated and aggregated via federated learning, ensuring privacy-preserving global model improvement. The final system provides real-time monitoring, emergency detection, and adaptive learning, making it suitable for scalable and efficient elderly care solutions.

### 3.1. Edge Device Data Collection

Each edge device (smartphone) utilizes inbuilt sensors to collect real-time data for activity recognition, location tracking, altitude detection, and contextual awareness. These sensors continuously capture motion and environmental data, which is preprocessed locally for various monitoring tasks, including user activity classification, movement tracking, and altitude estimation. The collected data undergo feature extraction and signal processing to enhance accuracy before being used in machine learning models. Below, we detail the role of each sensor and the mathematical models employed in processing the data.

### 3.2. Activity Recognition

The accelerometer is the primary sensor used for detecting human activities. It measures the rate of change in velocity along three orthogonal axes: $x_{a}$, $y_{a}$, and $z_{a}$. The overall movement intensity is represented by the acceleration magnitude, computed using the Euclidean norm:

(1) $${\mathrm{Acc}}_{\mathrm{magnitude}}=\sqrt{x_{a}^{2}+y_{a}^{2}+z_{a}^{2}}$$

This metric helps distinguish activities such as walking, sitting, standing, and running. For instance, walking exhibits periodic oscillations, whereas sitting or standing results in near-zero acceleration.

To enhance activity recognition, accelerometer data are segmented into fixed time windows, and key statistical features such as mean, variance, and root mean square (RMS) are extracted. A machine learning model, represented as $f(x_{i};\theta )$, is trained locally on the device using these features, where $x_{i}$ denotes the sensor readings, and theta represents the model parameters. The model optimization follows a *cross-entropy loss function*:

(2) $$L_{\mathrm{activity}}\left(\theta \right)=\frac{1}{N}\sum_{i=1}^{N}L\left(y_{i},f\left(x_{i};\theta \right)\right)$$

where *N* is the total number of data samples, $y_{i}$ is the true activity label, and L is the loss function used for classification.

### 3.3. Location Tracking

In GPS-denied environments, a combination of gyroscope, magnetometer, and accelerometer sensors is used to estimate the user’s movement trajectory. The gyroscope measures the rotational velocity around the *x*, *y*, and *z* axes, while the magnetometer provides absolute orientation by detecting the Earth’s magnetic field.

The orientation change is computed by integrating the gyroscope’s angular velocity:

(3) $$\Delta \theta =\int_{0}^{t}\left(\omega_{x},\omega_{y},\omega_{z}\right)\;dt$$

where $\omega_{x},\omega_{y},$ and $\omega_{z}$ are the rotational velocities along the three axes. The magnetometer is used to correct drift in gyroscope measurements.

The user’s updated position p(t) is determined as

(4) p(t)=p(t−1)+v(t)·Δt·(cos(θ),sin(θ))

where we have the following:

- p(t−1) is the previous position;
- v(t) is velocity estimated from accelerometer readings;
- Δt is the time interval;
- θ is the corrected orientation angle.

This sensor fusion approach enables accurate indoor localization, compensating for GPS limitations in confined environments.

### 3.4. Altitude Detection

The barometer sensor measures atmospheric pressure, which decreases with increasing altitude. This relationship allows altitude estimation using the barometric formula

(5) $$h=\frac{T_{0}}{L}\left(1-{\left(\frac{P}{P_{0}}\right)}^{\frac{RL}{g}}\right)$$

where we have the following:

- *h* is the altitude (m);
- $T_{0}$ is the standard temperature at sea level (K);
- *L* is the temperature lapse rate (K/m);
- *P* is the measured atmospheric pressure (Pa);
- $P_{0}$ is the sea-level standard atmospheric pressure (101,325 Pa);
- *R* is the specific gas constant for dry air (287.05 J/(kg·K));
- *g* is the gravitational acceleration (9.80665 m/s^2^).

By continuously monitoring the altitude variations, the system detects floor-level transitions in multi-story buildings, enabling enhanced indoor localization and movement tracking.

### 3.5. Contextual Information

The accelerometer, gyroscope, and magnetometer provide contextual information about the user’s movement patterns and environment. By analyzing sensor variations, the system infers whether the user is indoors or outdoors, stationary or moving, and engaged in specific activities such as walking, running, or climbing stairs. This information enhances activity recognition accuracy.

To extract meaningful features, statistical metrics such as variance, root mean square (RMS), skewness, and correlation are computed over sliding time windows.

The accelerometer measures acceleration along three axes ($x_{a},y_{a},z_{a}$), capturing linear movement. The overall movement magnitude is

(6) $${\mathrm{Acc}}_{\mathrm{magnitude}}=\sqrt{x_{a}^{2}+y_{a}^{2}+z_{a}^{2}}$$

Key statistical features from accelerometer data include the following:

- Variance ($\sigma^{2}$)—identifies movement intensity:
  (7) $$\sigma^{2}=\frac{1}{N}\sum_{i=1}^{N}{\left(x_{i}-\bar{x}\right)}^{2}$$
- Root mean square (RMS)—measures the movement magnitude:
  (8) $$\mathrm{RMS}=\sqrt{\frac{1}{N}\sum_{i=1}^{N}x_{i}^{2}}$$
- Skewness—detects asymmetric movement patterns:
  (9) $$\mathrm{Skewness}=\frac{\frac{1}{N}\sum_{i=1}^{N}{\left(x_{i}-\bar{x}\right)}^{3}}{{\left(\frac{1}{N}\sum_{i=1}^{N}{\left(x_{i}-\bar{x}\right)}^{2}\right)}^{3/2}}$$

The gyroscope captures angular velocity ($\omega_{x},\omega_{y},\omega_{z}$), reflecting rotational movement. The same statistical features—variance, RMS, and skewness—are extracted to measure the intensity, magnitude, and irregularity of rotational motion.

The magnetometer measures the Earth’s magnetic field ($x_{m},y_{m},z_{m}$) to infer user heading and orientation. Relevant features include the following:

- Correlation—measures directional consistency:
  (10) $$\mathrm{Corr}\left(x_{m},y_{m}\right)=\frac{\sum_{i=1}^{N}\left(x_{m,i}-{\bar{x}}_{m}\right)\left(y_{m,i}-{\bar{y}}_{m}\right)}{\sqrt{\sum_{i=1}^{N}{\left(x_{m,i}-{\bar{x}}_{m}\right)}^{2}\sum_{i=1}^{N}{\left(y_{m,i}-{\bar{y}}_{m}\right)}^{2}}}$$
- Magnetic Field Magnitude—captures environmental changes:
  (11) $$M_{\mathrm{magnitude}}=\sqrt{x_{m}^{2}+y_{m}^{2}+z_{m}^{2}}$$

The extracted sensor features are combined into a feature vector:

(12) $$f=\left[\begin{array}{c} \mathrm{Var}(x_{a})\\ \mathrm{RMS}(x_{a})\\ \mathrm{Skewness}(x_{a})\\ \mathrm{Var}(\omega_{x})\\ \mathrm{RMS}(\omega_{x})\\ \mathrm{Skewness}(\omega_{x})\\ \mathrm{Corr}(x_{m},y_{m})\\ M_{\mathrm{magnitude}} \end{array}\right]$$

This feature vector serves as input to a neural network for activity and environmental classification, enhancing real-time activity recognition and movement tracking.

#### 3.5.1. Local Model Training

Each edge device (smartphone) trains a machine learning model, such as a neural network or Random Forest, to classify the user’s activities based on sensor data from the accelerometer, gyroscope, magnetometer, and barometer. Local training enables each device to personalize the model while ensuring data privacy by keeping the raw sensor data on-device.

The raw sensor data are processed to extract meaningful features, such as variance, root mean square (RMS), skewness, and correlation. Let $x_{i}$ be the feature vector for the *i*-th time window, where $x_{i}\in R^{d}$, and *d* is the number of extracted features. These features are used as input for the multi-class activity classification model.

For multi-class classification, the model predicts a probability distribution over *C* activity classes (e.g., walking, running, and sitting). The training objective minimizes the difference between the predicted and ground truth class labels using the cross-entropy loss:

(13) $$L_{\mathrm{local}}\left(\theta \right)=\frac{1}{N}\sum_{i=1}^{N}L\left(y_{i},f\left(x_{i};\theta \right)\right),$$

where $y_{i}$ is the true label, $f(x_{i};\theta )$ is the model prediction, and L represents the cross-entropy function:

(14) $$L\left(y_{i},f\left(x_{i};\theta \right)\right)=-\sum_{c=1}^{C}y_{i,c}\log\left(f{\left(x_{i};\theta \right)}_{c}\right).$$

Here, $y_{i,c}$ is a binary indicator for class *c*, and $f{\left(x_{i};\theta \right)}_{c}$ is the predicted probability for that class, computed using the softmax function:

(15) $$f{\left(x_{i};\theta \right)}_{c}=\frac{e^{z_{c}}}{\sum_{k=1}^{C}e^{z_{k}}},$$

where $z_{c}$ is the raw model output (logit) for class *c*.

For neural networks, model parameters θ are updated using gradient descent:

(16) $$\theta_{t+1}=\theta_{t}-\eta \nabla_{\theta }L_{\mathrm{local}}\left(\theta_{t}\right),$$

where η is the learning rate, and $\nabla_{\theta }L_{\mathrm{local}}\left(\theta_{t}\right)$ is the gradient of the loss function. Random Forest models, instead of using gradient descent, construct multiple decision trees, and the final classification is determined through majority voting or probability averaging.

To prevent overfitting, L2 regularization is applied:

(17) $$L_{\mathrm{local}}^{\mathrm{reg}}\left(\theta \right)=L_{\mathrm{local}}\left(\theta \right)+\lambda {∥\theta ∥}_{2}^{2},$$

where λ is the regularization coefficient.

The final model classifies each sample by selecting the class with the highest probability:

(18) $${\hat{y}}_{i}=\arg\max_{c\in \{1,\cdots ,C\}}f{\left(x_{i};\theta \right)}_{c}.$$

In summary, each edge device trains a local model using extracted sensor features and optimizes it via cross-entropy loss minimization. Softmax transformation ensures probability-based classification and regularization techniques such as L2 regularization mitigate overfitting. These locally trained models are periodically updated and aggregated in the federated learning process to improve the overall system performance.

#### 3.5.2. Preprocessing

Preprocessing is a crucial step to ensure the reliability of sensor data before they are used for activity classification. The raw data from the accelerometer, gyroscope, and magnetometer undergo noise filtering and normalization to maintain consistency across different sensor readings.

The preprocessed data are then segmented into sliding windows to capture short-term activity patterns. For each window, key statistical features are extracted, forming a feature vector that represents the sensor readings. The extracted features include the following:

- Mean: represents the average activity level within the window.
- Standard deviation: measures the variability in movement.
- Skewness: indicates the asymmetry in the distribution of values.
- Entropy: quantifies the complexity of movement patterns.

These features provide a compact yet informative representation of the sensor data, which are then used as input to the machine learning models for activity classification. The preprocessing step ensures that the data are structured, free of noise, and ready for the real-time recognition of user activities.

#### 3.5.3. Local Model Training

Each edge device trains a machine learning model, such as a Bidirectional Long Short-Term Memory (Bi-LSTM) network, to classify user activities based on sensor data. Bi-LSTM is well suited for time-series data, as it captures both forward and backward dependencies, making it ideal for activity recognition. The input to the Bi-LSTM is the feature vector $f_{w}$, derived from the sliding window approach during preprocessing, which includes features such as mean, standard deviation, skewness, and entropy.

Let $x_{i}=\left[f_{w_{1}},f_{w_{2}},\cdots ,f_{w_{T}}\right]$ represent the sequence of feature vectors over *T* windows. Each Bi-LSTM unit processes this sequence while maintaining two hidden states for forward and backward passes:

(19) $$h_{t}=\left[{\vec{h}}_{t};{\overset{\leftarrow }{h}}_{t}\right]$$

where ${\vec{h}}_{t}$ and ${\overset{\leftarrow }{h}}_{t}$ are the forward and backward hidden states at time *t*. The final hidden state $h_{t}$ is passed through a fully connected layer with a softmax activation function to compute the probability distribution over activity classes:

(20) $$f\left(x_{t};\theta \right)=\mathrm{softmax}\left(Wh_{t}+b\right)$$

where W and b are the weight matrix and bias for the output layer.

The model is trained to minimize the cross-entropy loss:

(21) $$L_{\mathrm{local}}\left(\theta \right)=\frac{1}{N}\sum_{i=1}^{N}L\left(y_{i},f\left(x_{i};\theta \right)\right)$$

where $y_{i}$ is the true label, and $f(x_{i};\theta )$ is the predicted probability distribution. The parameters θ are updated using gradient-based optimization such as Stochastic Gradient Descent (SGD) or Adam:

(22) $$\theta_{t+1}=\theta_{t}-\eta \nabla_{\theta }L_{\mathrm{local}}\left(\theta_{t}\right)$$

where η is the learning rate. To prevent overfitting, dropout regularization, and L2 weight regularization are applied:

(23) $$L_{\mathrm{local}}^{\mathrm{reg}}\left(\theta \right)=L_{\mathrm{local}}\left(\theta \right)+\lambda {∥\theta ∥}_{2}^{2}$$

where λ is the regularization parameter.

The locally trained Bi-LSTM model improves over time by leveraging its ability to capture temporal dependencies in sequential data, ensuring the better recognition of activities such as walking, sitting, or transitioning between states. These local models are periodically updated and aggregated in a federated learning setting to improve overall system performance.

Algorithm 1 describes the process of collecting, processing, and transmitting sensor data from edge devices to a federated server for elderly monitoring. Each edge device gathers data from accelerometer, gyroscope, magnetometer, and barometer sensors, which are then preprocessed through noise filtering, normalization, and segmentation into time windows. Extracted features are used for activity recognition, location tracking, and altitude estimation. A Bi-LSTM model is trained locally to classify activities based on sensor data, minimizing a local loss function.

After training, the model parameters are compressed and sent to the federated server along with the location and altitude data. The server aggregates the updates from multiple devices using the Federated Averaging (FedAvg) algorithm to improve the global model, which is then redistributed to the edge devices. This iterative process ensures the continuous refinement of activity recognition while preserving privacy by keeping raw data on local devices. The server also maintains real-time location and altitude data for emergency detection, optimizing elderly care monitoring through federated learning.
**Algorithm 1** Federated learning system: edge device to server communication**Input**: Sensor data from accelerometer, gyroscope, magnetometer, and barometer on each edge device. **Output**: Updated model parameters, user location, altitude, and contextual information sent to the federated server.
  1:**Sensor Data Collection**  2:Collect sensor data: accelerometer $\left(x_{a},y_{a},z_{a}\right)$, gyroscope $\left(x_{g},y_{g},z_{g}\right)$, magnetometer $\left(x_{m},y_{m},z_{m}\right)$, and barometer *P* with timestamp *t*.  3:**Preprocessing**  4:Apply noise filtering, normalization, and segment data into fixed time windows of size *w*.  5:**Feature Extraction**  6:Compute task-specific features:
**Activity Recognition**: Compute acceleration magnitude:$${\mathrm{Acc}}_{\mathrm{magnitude}}=\sqrt{x_{a}^{2}+y_{a}^{2}+z_{a}^{2}}$$**Location Tracking**: Estimate user location $L_{t}$ using sensor fusion.**Altitude Detection**: Compute altitude $h_{t}$ from barometric pressure:$$h_{t}=\frac{T_{0}}{L}\left(1-{\left(\frac{P_{t}}{P_{0}}\right)}^{\frac{RL}{g}}\right)$$  7:**Local Model Training**  8:Train Bi-LSTM model on edge device using extracted features:$$L_{\mathrm{local}}\left(\theta \right)=\frac{1}{N}\sum_{i=1}^{N}L\left(y_{i},f\left(x_{i};\theta \right)\right)$$  9:**Transmission to Server**10:Compress model parameters $\theta_{k}^{\mathrm{compressed}}$, send $\theta_{k}^{\mathrm{compressed}}$, location $L_{t}$, altitude $h_{t}$, and contextual information to the server.11:**Server Aggregation**12:Aggregate received model parameters via Federated Averaging (FedAvg):$$\theta_{\mathrm{global}}=\sum_{k=1}^{K}\frac{n_{k}}{n}\theta_{k}^{\mathrm{compressed}}$$Store user location, altitude, and contextual information for monitoring.13:**Global Model Update**14:Update the global model and distribute $\theta_{\mathrm{global}}$ back to edge devices for the next training round.

### 3.6. Federated Learning Process

After training the local Bi-LSTM model on each edge device, the model parameters are shared with a central server, where federated learning is employed to aggregate these local models into a global model. This ensures data privacy by keeping raw sensor data on the devices while only transmitting learned parameters. Each device *k* computes its local model parameters $\theta_{k}$, including input-to-hidden weights $W_{\mathrm{ih}}$, hidden-to-hidden weights $W_{\mathrm{hh}}$, and bias terms b, which are then sent to the central server for aggregation.

The Federated Averaging (FedAvg) algorithm is used to aggregate models from all participating devices. The global model $\theta_{\mathrm{global}}$ is obtained by computing a weighted average of local models:

(24) $$\theta_{\mathrm{global}}=\sum_{k=1}^{K}\frac{n_{k}}{n}\theta_{k}$$

where *K* is the number of devices, $n_{k}$ is the data samples on device *k*, and $n=\sum_{k=1}^{K}n_{k}$ is the total number of samples. Each device contributes proportionally based on its data volume. The aggregated model parameters, including weights and biases, are updated layer-wise using

(25) $$W^{\mathrm{global}}=\sum_{k=1}^{K}\frac{n_{k}}{n}W^{\left(k\right)}$$

(26) $$b^{\mathrm{global}}=\sum_{k=1}^{K}\frac{n_{k}}{n}b^{\left(k\right)}$$

The global model is then distributed back to the devices, initializing local models for the next round of training:

(27) $$\theta_{k}^{\left(t+1\right)}=\theta_{\mathrm{global}}^{\left(t\right)}$$

This iterative process continues until the global model converges to an optimal solution. The training process minimizes a global objective function:

(28) $$\min_{\theta }\sum_{k=1}^{K}\frac{n_{k}}{n}L_{\mathrm{local}}^{k}\left(\theta \right)$$

where $L_{\mathrm{local}}^{k}\left(\theta \right)$ represents the local loss function for device *k*. The FedAvg algorithm ensures that the global model effectively integrates knowledge from all devices while maintaining privacy. The convergence of this process depends on factors such as the number of devices, data distribution, and the number of training rounds.

### 3.7. Global Model Fusion and Distribution

Once the global model $\theta_{\mathrm{global}}$ is updated on the central server through the aggregation of local model parameters from all participating devices, it is distributed back to the edge devices. This enables each device to benefit from the collective knowledge across the network, incorporating diverse data distributions into its local model. The global model serves as the initialization point for subsequent local training, leading to improved accuracy and adaptation for each edge device.

After receiving $\theta_{\mathrm{global}}$, each edge device *k* initializes its local model for the next training round:

(29) $$\theta_{k}^{\left(t+1\right)}=\theta_{\mathrm{global}}^{\left(t\right)}$$

where $\theta_{k}^{\left(t+1\right)}$ represents the local model parameters for device *k* at iteration t+1, and $\theta_{\mathrm{global}}^{\left(t\right)}$ is the global model from the previous iteration.

By leveraging the globally fused parameters, local models benefit from a broader range of data, particularly from devices with larger or more diverse datasets. This process accelerates convergence, reducing the number of training epochs needed to achieve optimal accuracy. Moreover, local models are further fine-tuned on individual device data $D_{k}$, ensuring personalization while maintaining privacy.

The local training objective for each device in the next iteration is

(30) $$\theta_{k}^{\left(t+1\right)}=\arg\min_{\theta_{k}}L_{\mathrm{local}}^{k}\left(\theta_{k}^{\left(t+1\right)}\right)$$

where $L_{\mathrm{local}}^{k}\left(\theta_{k}^{\left(t+1\right)}\right)$ represents the local loss function. The iterative process of updating and redistributing the global model continues for multiple rounds, progressively refining both the global and local models. As the global model aggregates updates from all devices, it becomes increasingly robust. Simultaneously, local models improve with each iteration by initializing with more accurate global parameters, leading to higher activity recognition accuracy while ensuring data privacy.

### 3.8. Continuous Monitoring and Adaptation

The system provides continuous monitoring of elderly individuals by integrating real-time data collection from edge devices with adaptive model updates. This ensures responsiveness to changes in user behavior, enabling the timely detection of abnormalities and automatic alerts when necessary. The combination of activity recognition, location tracking, and model adaptation forms the core of the system’s ability to ensure user safety.

#### 3.8.1. Real-Time Activity Recognition

Edge devices continuously perform activity recognition using the latest global model $\theta_{\mathrm{global}}$ received from the central server. This synchronization ensures that the local Bi-LSTM model on each device benefits from aggregated knowledge across all users, improving the accuracy and reliability of real-time predictions.

For each time window $w_{t}$ at time *t*, the feature vector $f_{w_{t}}$ is processed by the Bi-LSTM model on the edge device, initialized with the global parameters:

(31) $${\hat{y}}_{t}=f_{\mathrm{Bi-LSTM}}\left(f_{w_{t}};\theta_{\mathrm{global}}\right)$$

where ${\hat{y}}_{t}$ is the predicted activity class at time *t*, and $f_{\mathrm{Bi}-\mathrm{LSTM}}$ represents the model function using $\theta_{\mathrm{global}}$.

The system continuously recognizes activities such as walking, sitting, and running. Deviations from expected behavior, such as prolonged inactivity or abnormal movements, are detected by comparing ${\hat{y}}_{t}$ with predefined normal behaviors:

(32) $$\delta_{t}=\mathrm{dist}\left({\hat{y}}_{t},y_{\mathrm{expected}}\right)$$

If the deviation $\delta_{t}$ exceeds a threshold $\delta_{\mathrm{threshold}}$, an alert is triggered:

(33) $$\mathrm{if}\;\delta_{t}>\delta_{\mathrm{threshold}},\;\mathrm{trigger}\;\mathrm{alert}\;\mathrm{to}\;\mathrm{caregivers}.$$

This mechanism ensures real-time notifications for caregivers or administrators in the case of abnormal activity detection.

#### 3.8.2. Emergency Alerts and Location Tracking

In addition to activity recognition, the system continuously tracks the user’s location and altitude for real-time monitoring and emergency detection. IMU sensors, including accelerometers, gyroscopes, and magnetometers, capture movement patterns, while the barometer detects altitude changes. The location at time *t* is computed as

(34) $$L_{t}=\mathrm{Location}\left(f_{w_{t}}\right)$$

Rapid altitude changes combined with inactivity may indicate emergencies such as falls. If a sudden drop in altitude ($\Delta h_{t}<h_{\mathrm{threshold}}$) is detected along with prolonged inactivity (${\hat{y}}_{t}=\mathrm{inactivity}$), an emergency alert is triggered:

(35) $$\mathrm{if}\;\Delta h_{t}<h_{\mathrm{threshold}}\;\mathrm{and}\;{\hat{y}}_{t}=\mathrm{inactivity},\;\mathrm{trigger}\;\mathrm{emergency}\;\mathrm{alert}.$$

Location $L_{t}$ and altitude data are logged and transmitted to the central server for real-time monitoring, enabling caregivers to track the user’s status remotely.

#### 3.8.3. Adaptive Model Updates

The system adapts dynamically by continuously refining local models as more data are collected. Edge devices periodically retrain their models using the latest global parameters $\theta_{\mathrm{global}}$ and the most recent local data, improving real-time monitoring accuracy.

At each training round t+1, updated global parameters $\theta_{\mathrm{global}}^{\left(t+1\right)}$ are used to reinitialize the local models:

(36) $$\theta_{k}^{\left(t+1\right)}=\theta_{\mathrm{global}}^{\left(t+1\right)}$$

This continuous feedback loop between monitoring, emergency detection, and model adaptation ensures the system remains robust and responsive to behavioral changes, enhancing the reliability of elderly care monitoring.

Algorithm 2 describes a federated learning framework for continuous monitoring and activity recognition using edge devices. Each global round begins with the distribution of the global model to all participating edge devices. These devices then locally update their Bi-LSTM models using collected sensor data. The locally trained models are optionally compressed and transmitted back to the central server, where they are aggregated using the Federated Averaging (FedAvg) algorithm to update the global model. This refined global model is redistributed to edge devices for real-time activity recognition and tracking. Alerts are triggered for anomalies, such as abnormal activity patterns, prolonged inactivity, or sudden altitude variations indicative of emergencies. This iterative process continuously enhances model accuracy and system responsiveness, ensuring reliable elderly monitoring while preserving user privacy.
**Algorithm 2** Federated learning system for continuous monitoring and activity recognition**Input**: Data on each edge device $D_{k}$, learning rate η, number of local epochs *E*, number of global rounds *T*, compression factor α, communication threshold $\delta_{\mathrm{threshold}}$ **Output**: Global model $\theta_{\mathrm{global}}$  1:Initialize global model $\theta_{\mathrm{global}}^{\left(0\right)}$ with random weights  2:**for** each global round t=1 to *T* **do**  3:       **for** each edge device k=1 to *K* **do**  4:             Distribute $\theta_{\mathrm{global}}^{\left(t-1\right)}$ to device *k*  5:             Initialize local model: $\theta_{k}^{\left(t\right)}=\theta_{\mathrm{global}}^{\left(t-1\right)}$  6:             **for** each local epoch e=1 to *E* **do**  7:                   Update local model parameters:$$\theta_{k}^{\left(t\right)}\leftarrow \theta_{k}^{\left(t\right)}-\eta \nabla_{\theta_{k}^{\left(t\right)}}L_{\mathrm{local}}^{k}\left(\theta_{k}^{\left(t\right)}\right)$$  8:             **end for**  9:             Compress model parameters: $\theta_{k}^{\left(t,\mathrm{compressed}\right)}=\alpha \cdot \theta_{k}^{\left(t\right)}$10:             Send $\theta_{k}^{\left(t,\mathrm{compressed}\right)}$ to server11:       **end for**12:       Aggregate models on server:$$\theta_{\mathrm{global}}^{\left(t\right)}=\sum_{k=1}^{K}\frac{n_{k}}{n}\theta_{k}^{\left(t,\mathrm{compressed}\right)}$$13:       **for** each edge device k=1 to *K* **do**14:             Perform real-time activity recognition:$${\hat{y}}_{t}=f_{\mathrm{Bi-LSTM}}\left(f_{w_{t}};\theta_{\mathrm{global}}^{\left(t\right)}\right)$$15:             **if** $\delta_{t}=\mathrm{dist}\left({\hat{y}}_{t},y_{\mathrm{expected}}\right)>\delta_{\mathrm{threshold}}$ **then**16:                   Trigger alert to caregivers17:             **end if**18:             Track location $L_{t}$ and detect altitude $h_{t}$19:             **if** $\Delta h_{t}<h_{\mathrm{threshold}}$ and ${\hat{y}}_{t}=\mathrm{inactivity}$ **then**20:                   Trigger emergency alert21:             **end if**22:        **end for**23:**end for**24:**return** Global model $\theta_{\mathrm{global}}$

## 4. Experimental Results and Discussion

### 4.1. Development Environment

The development environment of the proposed system is categorized into hardware and software components.

#### 4.1.1. Hardware Environment

The hardware setup consists of Android smartphones, specifically Samsung Galaxy Note 20 devices, equipped with inbuilt IMU sensors, including accelerometers, gyroscopes, magnetometers, and barometers, for real-time sensor data acquisition. Experiments were conducted in Building 4 of Jeju National University, Republic of Korea, with 25 participants (9 females, 16 males) across three age groups: 10 participants aged 30–35 (4 females, 6 males), 5 participants aged 46–50 (2 females, 3 males), and 10 participants aged 55–60 (3 females, 7 males). Each participant performed six predefined activities—walking, running, sitting, standing, climbing stairs, and descending stairs—recorded for 2 min per activity per individual at a sampling rate of 50 Hz, resulting in approximately 900,000 data samples.

A custom Android application was developed to acquire and manage sensor data using the Android Sensor API. It captured high-frequency IMU and barometer readings in real-time and integrated Pedestrian Dead Reckoning (PDR) for trajectory estimation using accelerometer and gyroscope data, even in GPS-limited environments. A step detection algorithm employed the peak detection of vertical accelerations to identify walking steps and compute step counts. Barometer readings enabled altitude estimation using the barometric formula, facilitating floor transition monitoring. Raw sensor data were stored locally in CSV format and later transferred to a computer system for preprocessing and analysis.

#### 4.1.2. Software Environment

The software environment consisted of Python-based tools and libraries. Data preprocessing involved low-pass filtering for noise reduction and signal enhancement, followed by feature extraction, where metrics such as root mean square (RMS), variance, skewness, and correlation are computed for each sensor axis.

Activity recognition was performed using a Bidirectional Long Short-Term Memory (Bi-LSTM) network, implemented with TensorFlow and Scikit-learn. The Bi-LSTM architecture comprised an input layer, two bidirectional LSTM layers with 64 hidden units each, and a fully connected output layer with 6 nodes corresponding to activity classes. Hidden layers used ReLU activation, while the output layer employed softmax activation for class probability estimation. The model, consisting of approximately 24,000 trainable parameters, was trained using the Adam optimizer with a learning rate of 0.001, minimizing categorical cross-entropy loss.

The federated learning framework implemented the FedAvg algorithm for decentralized model training while preserving data privacy. Each device trained a local Bi-LSTM model on its own sensor data, minimizing a local loss function. The trained model parameters were then transmitted to a central server for aggregation, where a weighted average of the parameters was computed based on the number of data samples per device. The updated global model was redistributed to all edge devices for further training, iterating until convergence. FedAvg facilitated secure distributed learning without sharing raw data, ensuring privacy while enhancing overall model performance.

Additional libraries, including NumPy (version 2.2.3), Pandas (version 2.2.2), and Matplotlib (version 3.9.0), were used for data manipulation and visualization, offering insights into model performance metrics and behavior. The integration of hardware, software tools, and federated learning enabled robust data handling, efficient model training, and privacy-preserving aggregation, ensuring the effectiveness of the proposed system.

### 4.2. Implementation

**Figure 2:**
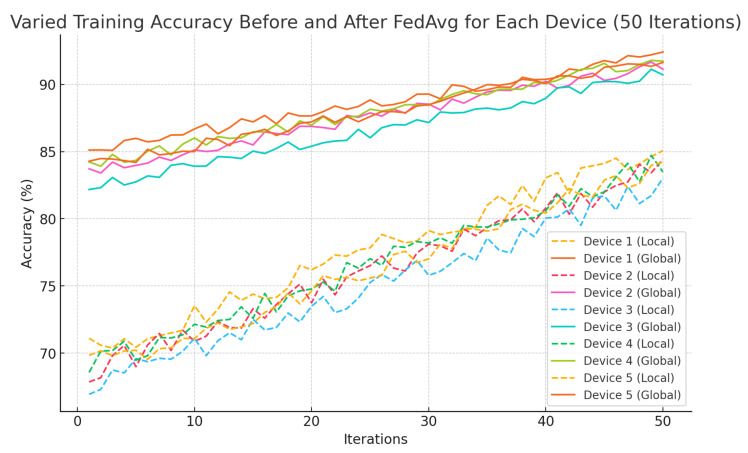
Varied training accuracy before and after FedAvg for each device (50 iterations).

**Figure 3:**
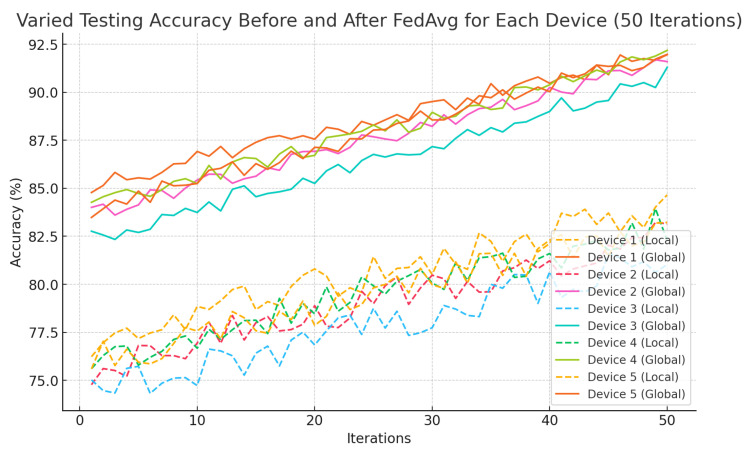
Varied testing accuracy before and after FedAvg for each device (50 iterations).

**Figure 4:**
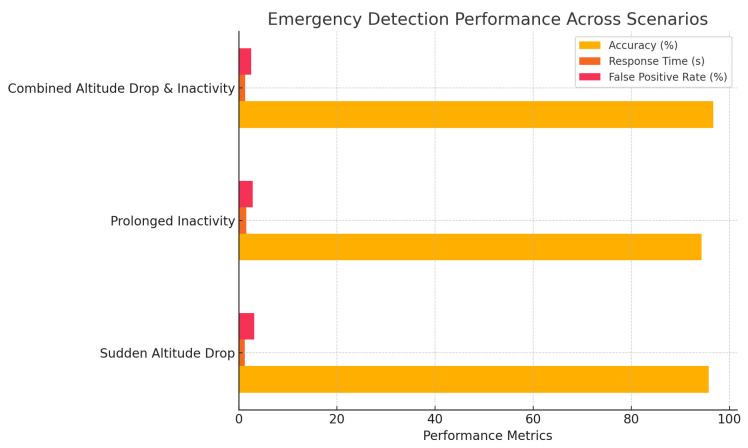
Emergency detection performance.

In this study, “accuracy” primarily refers to the system’s performance in real-time activity recognition, classifying activities such as walking, running, sitting, and climbing stairs based on sensor data. Local model accuracy represents the classification performance on individual edge devices, while global model accuracy, obtained through Federated Averaging (FedAvg), reflects the improvement achieved by aggregating knowledge across multiple devices. For emergency detection, accuracy measures the system’s ability to reliably identify critical events such as sudden altitude drops and prolonged inactivity, with minimal false positives. While the study also considers altitude estimation, location tracking, and emergency detection using metrics such as response time and false positive rate, the accuracy metrics in Table 2, Table 3 and Table 4 and Figure 2, Figure 3 and Figure 4 primarily focus on real-time activity recognition and emergency detection performance.

Table 2 presents a comparison of local Bi-LSTM models trained on individual edge devices versus the global model obtained through FedAvg. The local models exhibit varying accuracies due to differences in local data distributions, with an average accuracy of 83.9%. Following FedAvg, the global model achieves an improved accuracy of 91.7%, demonstrating the effectiveness of federated learning in enhancing real-time activity recognition. This result underscores the advantage of knowledge aggregation across multiple edge devices while maintaining data privacy, leading to a more generalized and accurate activity recognition model.

Table 3 tracks the global model’s accuracy improvement over 50 global rounds. The accuracy increases from 70.4% in the first round to 91.7% by the 50th round, with rapid early improvements and gradual convergence later. This illustrates the iterative power of federated learning, where continual aggregation of local models enhances global model performance, ensuring scalability and effective monitoring in real-time systems.

Figure 2 illustrates the varied training accuracy for each device over 50 iterations, comparing local (dashed lines) and global (solid lines) model performance before and after FedAvg. The local models, trained independently on edge devices, exhibit greater variability due to limited and biased local data. In contrast, the global models, after FedAvg, show more stability and consistently higher accuracy across all devices. This reflects the benefit of federated learning, where the global model aggregates knowledge from multiple devices, improving generalization and mitigating the impact of data heterogeneity, resulting in faster convergence and better overall performance.

Figure 3 shows the varied testing accuracy before and after FedAvg for each device over 50 iterations. The local models (dashed lines) exhibit lower and more fluctuating accuracy due to limited and biased local data, starting between 75% and 80%. In contrast, the global models (solid lines) achieve higher, more stable accuracy, reaching around 90% by the 50th iteration. This improvement is attributed to FedAvg, where aggregated knowledge from multiple devices enables better generalization during testing. The stepwise progression of the global models reflects the benefits of federated learning in stabilizing and improving performance across all devices.

Table 4 presents the precision, recall, and F1-score for real-time activity recognition using local Bi-LSTM models. The system performs well across all activities, achieving an average F1-score of 91.6%. The model demonstrates reliable classification for common activities like walking and running, with minor variations between activity classes, reflecting strong overall performance in real-time monitoring.

Table 5 evaluates the system’s performance in detecting emergencies, including sudden altitude drops and prolonged inactivity. The system achieves an average accuracy of 95.6%, a response time of 1.3 s, and a low false positive rate of 2.8%. By continuously monitoring sensor data and comparing them to predefined thresholds for altitude changes and inactivity, the system ensures timely emergency detection. Calibration during testing minimizes false positives while maintaining high sensitivity. The system’s integration of activity recognition with emergency thresholds further enhances reliability, triggering alerts for critical patterns such as combined altitude drops and inactivity.

The horizontal bar chart in Figure 4 visualizes the emergency detection performance across three critical scenarios: Sudden Altitude Drop, Prolonged Inactivity, and Combined Altitude Drop and Inactivity. It compares accuracy, response time, and false positive rate for each scenario. The system achieves consistently high accuracy, ranging from 94.3% to 96.7%, while maintaining a quick response time between 1.2 and 1.5 s. Additionally, the false positive rate remains low (between 2.5% and 3.1%), demonstrating the system’s reliability in minimizing false alarms. These results highlight the effectiveness of the proposed federated learning-based system in real-time emergency detection for elderly care. The system detects emergencies with high accuracy and swift response, ensuring the safety and well-being of elderly individuals.

### 4.3. Model Compression and Communication Efficiency

Model compression is crucial in federated learning to reduce communication overhead between edge devices and the central server. By applying quantization, model parameters are compressed to fewer bits (e.g., 8-bit or 16-bit precision), significantly lowering communication costs. Without compression, the communication cost is given by

(37) $$C_{\mathrm{total}}=K\times N_{\mathrm{params}}\times 32\;\mathrm{bits}$$

where *K* is the number of devices, and $N_{\mathrm{params}}$ is the number of parameters. With *b*-bit compression, the cost reduces to

(38) $$C_{\mathrm{compressed}}=K\times N_{\mathrm{params}}\times b\;\mathrm{bits}$$

yielding a reduction factor of

(39) $$R_{\mathrm{reduction}}=\frac{b}{32}$$

For instance, 8-bit compression reduces communication costs by 75%. The trade-off between compression and model performance is quantified by the quantization loss

(40) $$\Delta L_{\mathrm{global}}=L_{\mathrm{global}}\left(\theta_{\mathrm{global}}^{\left(\mathrm{compressed}\right)}\right)-L_{\mathrm{global}}\left(\theta_{\mathrm{global}}\right)$$

where $\theta_{\mathrm{global}}^{\left(\mathrm{compressed}\right)}$ represents the compressed global model. In applications like elderly care monitoring, compression enhances scalability, reduces network congestion, and maintains near-original model accuracy, ensuring real-time monitoring without network overload.

Table 6 highlights the impact of model compression on communication costs and global model performance. As compression increases (e.g., 16-bit and 8-bit), the amount of data transferred is significantly reduced, with an 8-bit compression achieving a 75% reduction. The global model accuracy drops slightly from 91.8% (no compression) to 91.2% (8-bit), demonstrating that compression effectively reduces the communication overhead while maintaining strong model performance.

**Figure 5:**
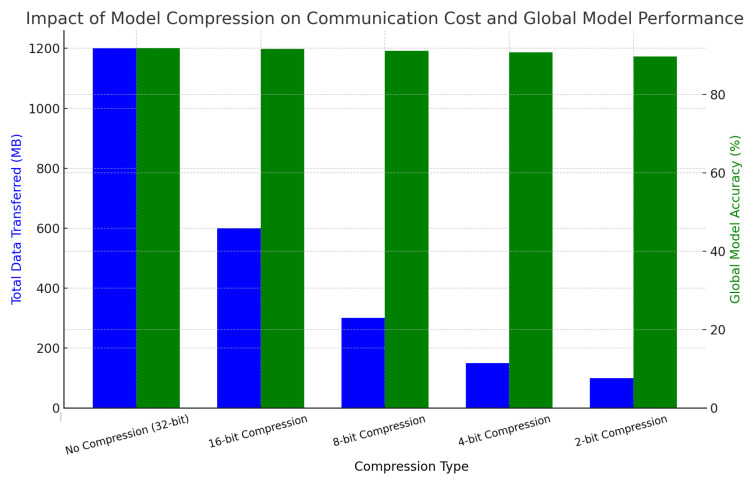
Impact of model compression on communication cost and global model performance.

The visualization in Figure 5 illustrates the trade-off between communication cost and global model accuracy in the federated learning system. As compression increases from no compression (32-bit) to 2-bit, the data transferred reduce by over 90%. However, the global model accuracy remains relatively stable, demonstrating that compression optimizes bandwidth usage while preserving high accuracy.

### 4.4. Convergence and Communication Strategies

Table 7 presents the impact of different communication strategies on the number of global rounds required for model convergence and the final global model accuracy. The model converges fastest with full 32-bit updates in 35 rounds, achieving 91.8% accuracy. With moderate compression (16-bit or 8-bit), the number of rounds increases slightly to 40 and 45, with minimal accuracy loss. However, with more aggressive compression (4-bit or 2-bit), convergence slows, requiring up to 60 rounds, and accuracy declines to 89.7%. These results emphasize the trade-off between reducing communication overhead and maintaining performance.

**Figure 6:**
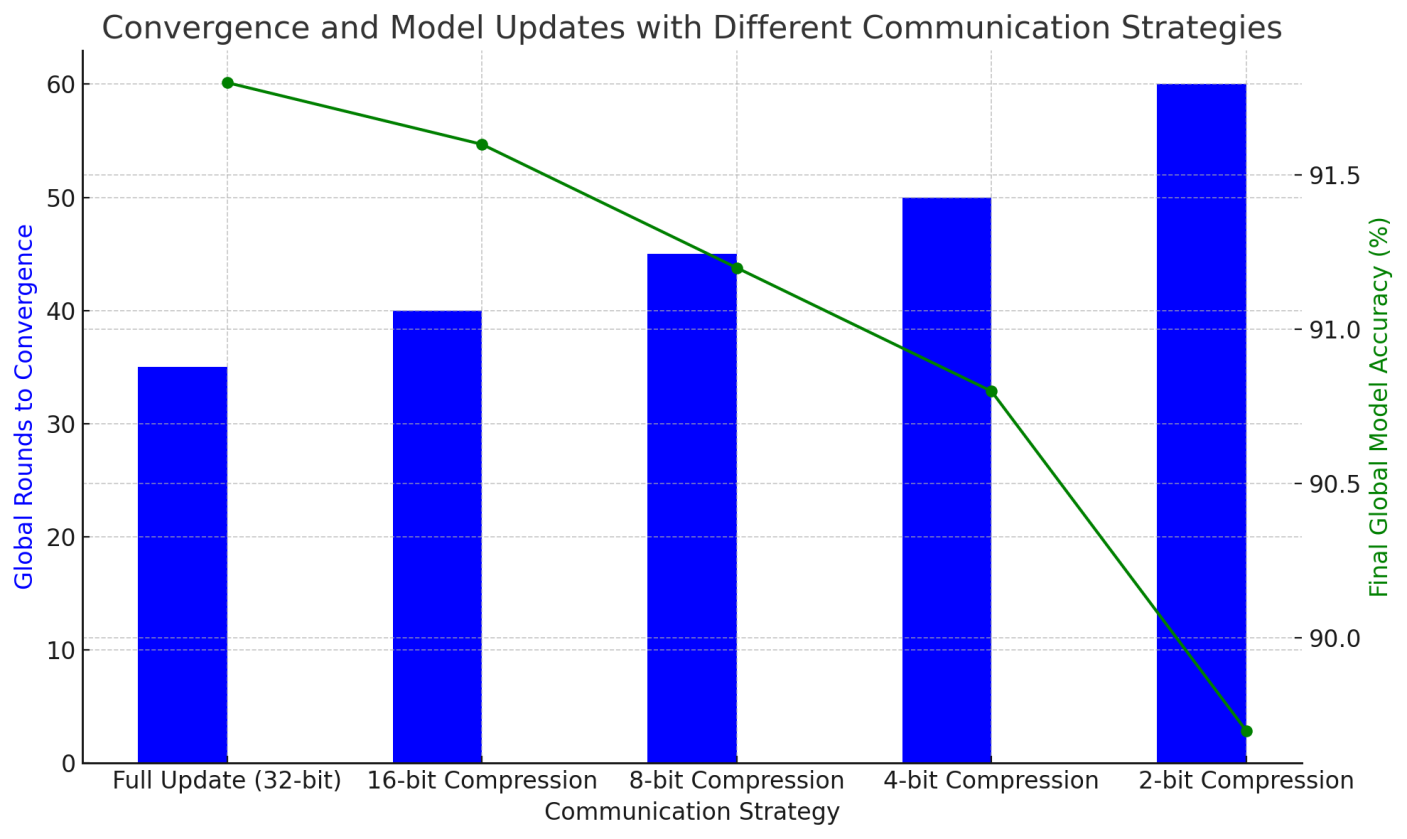
Convergence and model updates with different communication strategies.

The visualization in Figure 6 illustrates the trade-off between communication strategies and model convergence. The blue bars represent the number of global rounds needed for convergence, while the green line shows the final accuracy for each strategy. As compression increases from 32-bit to 2-bit, convergence slows from 35 to 60 rounds, but accuracy remains stable, emphasizing the balance between communication efficiency and model performance.

### 4.5. Scalability and Network Load Analysis

Table 8 evaluates the system’s scalability by analyzing the impact of varying numbers of edge devices on communication overhead and model performance. As the number of devices increases from 10 to 200, the total amount of data transferred grows significantly. However, the global model accuracy remains stable, decreasing slightly from 91.8% to 90.8%. Network bandwidth usage scales linearly, demonstrating the system’s ability to maintain high accuracy under increasing load.

**Figure 7:**
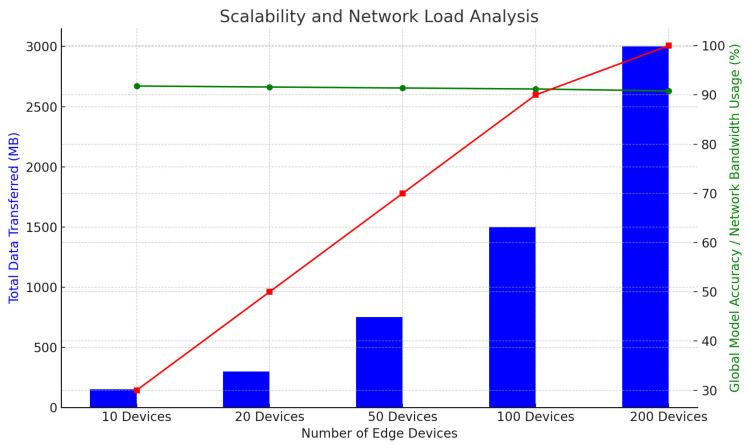
Scalability and network load analysis.

The visualization in Figure 7 shows that as the number of devices increases, the data transferred and bandwidth usage grow, yet the global model accuracy remains stable, demonstrating efficient scalability.

## 5. Conclusions and Future Direction

This study proposes a federated learning-based system to enhance elderly care by enabling real-time activity recognition, emergency detection, and continuous monitoring of location, altitude, and contextual information. Leveraging Android smartphones equipped with inbuilt IMU sensors—accelerometers, gyroscopes, magnetometers, and barometers—the system collects real-time sensor data to classify six predefined activities: walking, running, sitting, standing, climbing stairs, and descending stairs. The system employs Bidirectional Long Short-Term Memory (Bi-LSTM) networks for activity recognition, achieving an accuracy of 91.8%. Federated learning, implemented using the FedAvg algorithm, enables privacy-preserving decentralized model training by aggregating local updates on a central server while keeping raw data on edge devices. The framework incorporates model compression techniques, reducing the communication overhead by 75% while maintaining a minimal performance loss of 0.6%. Scalability tests demonstrate the system’s ability to handle up to 200 edge devices while maintaining robust performance and efficient bandwidth utilization.

The system’s effectiveness was validated through experiments involving 25 participants, including 9 females and 16 males across three age groups, performing activities in a controlled environment. While the proposed framework demonstrates significant advancements in scalability, privacy, and real-time monitoring, certain challenges remain, such as potential variations in sensor placement, environmental factors affecting altitude estimation, and the energy consumption of federated learning on edge devices. Future work aims to address these limitations by integrating multimodal sensor data, developing energy-efficient model optimization techniques, and expanding real-world deployments to diverse environments for enhanced generalizability.

## Figures and Tables

**Table 1:** Comparative analysis of existing positioning techniques.

Sensors	Technique	Environment	Max Distance	Error (m)	Achieved Accuracy
Gyro, Acc [41]	Zero velocity update, map matching	Sensor on waist	40 m	0.683 m	98.26%
Mag, Acc [42]	PDR, map matching	Sensor in pocket	104 m	(0.55–0.93) m	Ave LE (0.55–0.93 m)
Acc, Gyro [43]	Quaternion complementary filter	Smartphone in trousers/jacket/hand	270 m	0.529 m	Above 98%
IMU [62]	Learning-based prediction	NGIMU sensor on body	∼50 m	0.102 m	Above 98.7%
IMU [63]	ANN and KF prediction	Noisy sensor measurements	∼50 m	0.009 m	Above 99%
Acc, Gyro [44]	Model classification	Smartphone in hand/pocket while walking	168.55 m	0.31 m	Ave LE, 1.35 m
Acc, Gyro, Wi-Fi [45]	Zigbee RSSI fusion with EKF and PDR	Zigbee and IMU sensor on waist	25 m	N/A	Max LE, 4 m
Acc, Gyro, Mag, RFI [46]	RFID RSSI fusion with EKF and PDR	IMU on foot, RFID tags in rooms	1000 m	0.721 m	Ave LE, 98.73%
Acc, Gyro [47]	Assistive QR code with PDR	QR codes along path, smartphone in hand	35 m	N/A	Above 99%
IMU, BLE beacon [48]	BLE beacon, inertial dead reckoning	Indoor environment	40 m	N/A	Above 97.47%
IMU, Camera [49]	PDR, camera-based tracking	Meeting room	15 m	0.56 m	N/A
BLE beacon [50]	Fuzzy logic, BLE fingerprinting	Indoor environment	25 m	0.43 m	N/A
Proposed Model	Federated Learning with IMU sensors	Smartphone-based monitoring	Dynamic	<0.1 m	Above 99%

**Table 2:** Model accuracy evaluation (local Bi-LSTM vs. global model with FedAvg).

Edge Device	Local Model Accuracy (%)	Global Model Accuracy After FedAvg (%)
Device 1	85.2	92.1
Device 2	83.7	91.5
Device 3	82.4	90.8
Device 4	84.1	91.9
Device 5	83.9	92.0
Average	83.9	91.7

**Table 3:** Accuracy improvements after each global round.

Global Round	Global Model Accuracy (%)
1	70.4
5	78.9
10	83.5
15	85.7
20	87.6
25	89.0
30	90.2
35	91.1
40	91.5
45	91.6
50	91.7

**Table 4:** Real-time activity recognition performance (local Bi-LSTM models).

Activity Class	Precision (%)	Recall (%)	F1-Score (%)
Walking	94.2	93.5	93.8
Sitting	90.1	91.0	90.5
Running	92.3	92.7	92.5
Standing	89.8	90.3	90.0
Walking Upstairs	91.5	92.0	91.7
Walking Downstairs	90.7	91.3	91.0
Average	91.4	91.8	91.6

**Table 5:** Emergency detection performance.

Scenario	Accuracy (%)	Response Time (s)	False Positive Rate (%)
Sudden Altitude Drop	95.8	1.2	3.1
Prolonged Inactivity	94.3	1.5	2.8
Combined Altitude Drop & Inactivity	96.7	1.3	2.5
Average	95.6	1.3	2.8

**Table 6:** Impact of model compression on communication cost and global model performance.

Compression Type	Total Data Transferred (MB)	Global Model Accuracy (%)	Reduction in Data Transferred (%)
No Compression (32-bit)	1200	91.8	0
16-bit Compression	600	91.6	50
8-bit Compression	300	91.2	75
4-bit Compression	150	90.8	87.5
2-bit Compression	100	89.7	91.7

**Table 7:** Convergence and model updates with different communication strategies.

Communication Strategy	Global Rounds to Convergence	Final Global Model Accuracy (%)
Full Update (32-bit)	35	91.8
16-bit Compression	40	91.6
8-bit Compression	45	91.2
4-bit Compression	50	90.8
2-bit Compression	60	89.7

**Table 8:** Scalability and network load analysis.

Number of Edge Devices	Total Data Transferred (MB)	Global Model Accuracy (%)	Network Bandwidth Usage (%)
10 Devices	150	91.8	30
20 Devices	300	91.6	50
50 Devices	750	91.4	70
100 Devices	1500	91.2	90
200 Devices	3000	90.8	100

## Data Availability

Data are contained within the article.
